# Density-Dependent Plant–Plant Interactions Triggered by Grazing

**DOI:** 10.3389/fpls.2019.00876

**Published:** 2019-07-05

**Authors:** András Kelemen, Csaba Tölgyesi, Orsolya Valkó, Balázs Deák, Tamás Miglécz, Réka Fekete, Péter Török, Nóra Balogh, Béla Tóthmérész

**Affiliations:** ^1^MTA’s Post Doctoral Research Program, MTA TKI, Budapest, Hungary; ^2^Department of Ecology, University of Debrecen, Debrecen, Hungary; ^3^Department of Ecology, Faculty of Science and Technology, University of Szeged, Szeged, Hungary; ^4^MTA-DE Lendület Seed Ecology Research Group, Debrecen, Hungary; ^5^MTA-DE Biodiversity and Ecosystem Services Research Group, Debrecen, Hungary; ^6^Department of Botany, University of Debrecen, Debrecen, Hungary; ^7^MTA-DE Lendület Functional and Restoration Ecology Research Group, Debrecen, Hungary; ^8^Juhász Nagy Pál Doctorate School, University of Debrecen, Debrecen, Hungary

**Keywords:** biotic refuge, cattle grazing, competition, density-gradient, disturbance, facilitation, habitat heterogeneity

## Abstract

Plant species performance in rangelands highly depends on the effect of grazing and also on the occurrence of unpalatable benefactor species that can act as biotic refuges protecting neighboring plants from herbivores. The balance between facilitation and competition may changes with the benefactor density. Despite the high number of studies on the role of biotic refuges, the density dependent effects of unpalatable herbaceous plants on the performance of other species, and on the habitat heterogeneity of rangelands are still unclear. Therefore, we performed a study to test the following hypotheses: (i) Performances of understory species follow a humped-back relationship along the density gradient of the unpalatable benefactor species. (ii) Small-scale heterogeneity of the vegetation decreases with increasing benefactor density. We studied meadow steppes with medium intensity cattle grazing in Hungary. We surveyed understory species’ performance (number of flowering shoots and cover scores) along the density gradient of a common, native unpalatable species (*Althaea officinalis*). Our findings supported both hypotheses. We found unimodal relationship between the benefactor cover and both the flowering success and richness of understory species. Moreover, small-scale heterogeneity declined with increasing benefactor cover. In this study we detected a humped-back pattern of facilitation along the density gradient of an herbaceous benefactor in pastures. Indeed, this pattern was predictable based on such conceptual models like “consumer pressure-abiotic stress model,” “humped-back model,” “intermediate disturbance hypothesis,” and “disturbance heterogeneity model”; but until now the validity of these relationships has not been demonstrated for herbaceous species. By the demonstration of this effect between herbaceous species we can better forecast the responses of grasslands to changes in management.

## Introduction

There are both positive and negative plant-plant interactions in community assembly and their net effect may depend on the disturbance regime, including grazing (Smit et al., 2009). “Consumer pressure-abiotic stress model” hypothesized that facilitation between neighboring plants is likely present not only in abiotically stressed habitats (as predicted by “stress gradient hypothesis”), but also in communities influenced by consumer pressure (Bertness and Callaway, 1994; Hambäck and Beckerman, 2003). The general explanation of this model is that unpalatable plants (benefactors) act as biotic refuges by protecting neighboring plants from being eaten. The overwhelming majority of publications suggested that this facilitative effect of unpalatable plants has a maximum at moderate grazing pressure (Bossuyt et al., 2005; Smit et al., 2009). The balance between facilitation and competition in pasture vegetation may also change with the density of the benefactor (Bossuyt et al., 2005; Soliveres and Eldridge, 2014; Saixiyala et al., 2017). Increasing density of large-sized plants can cause humped-shape pattern of facilitation (Kesting et al., 2015). According to the “stress gradient hypothesis,” the ascending part of the curve could be explained by an ameliorated microenvironment (i.e., higher soil moisture and a more temperate microclimate; Maestre and Cortina, 2004; Kelemen et al., 2015b; Allegrezza et al., 2016). Moreover, as predicted by the “consumer pressure-abiotic stress model,” the shelter effect can also support the intensification of positive interactions in pastures (Soliveres and Eldridge, 2014). At the same time, the “shifting limitations hypothesis” (see Gibson, 2009) predicts that a decline of positive interactions is expected at high densities of large-sized plants due to their increased resource uptake and shading. The majority of these models stem from Grime’s “humped-back model” (Grime, 1973) which described a unimodal relationship between species richness (y-axis) and habitat productivity (*x*-axis). In this model x-axis includes two gradients from left to right: (i) increasing competition and (ii) decreasing intensity of stress and/or disturbance (e.g., grazing). Based on these considerations, we predict that the maximum level of facilitation in pastures occurs at medium benefactor density. Papers that studied grazing-mediated density dependence of plant interactions between herbaceous species have reported increasing (McNaughton, 1978; Bossuyt et al., 2005) or decreasing (Koyama et al., 2015) facilitation with the increasing benefactor density. Despite the theoretical predictions, humped-back pattern of facilitation in pastures along the density gradient of an unpalatable herbaceous species has not been reported.

Besides influencing species performances, unpalatable plants can also affect ecological processes by altering small-scale heterogeneity of pasture vegetation (Tilman, 1982). The disturbance by grazers can be the key factor in the governance of competition-colonization trade-offs, which is responsible for the support of the habitat heterogeneity (Valladares et al., 2015; Pásztor et al., 2016). In line with the “disturbance heterogeneity model,” most studies have reported that the highest level of habitat heterogeneity is typical at medium grazing pressure (Kolasa and Rollo, 1991; Török et al., 2016). The density of unpalatable plants can influence grazing pressure, because areas with a higher density of the benefactors are visited less frequently by the grazers. Therefore, grazing intensity decreases with an increasing benefactor density, so in pastures with medium grazing intensity (such as our study sites) we expect that habitat heterogeneity decreases with the increasing abundance of benefactors.

We aimed to fit in a new puzzle piece in the complex picture of the grazing-mediated plant interactions. Therefore, we studied the changes of plant–plant interactions and small-scale habitat heterogeneity along the density gradient of a common, native unpalatable species (*Althaea officinalis*) in mesic grasslands managed by medium intensity grazing. In this vein, we tested the following hypotheses: (i) Flowering success and species richness of understory species follow humped-back curve along the gradient of benefactor density. (ii) Small-scale heterogeneity decreases with increasing benefactor density.

## Materials and Methods

### Study Area and Sampling

The study area is located in Central Hungary (coordinates for the center: 46°46′N, 19°22′E). The climate in the region is continental, the mean annual temperature is 10°C and the mean annual precipitation is 520 mm (Vadász et al., 2016). The landscape covered by thousands of hectares pristine grasslands, most of which are perennial-dominated meadow steppes (for the detailed information of their species pool and dominance structure see Supplementary Appendix S1). The studied meadow steppes are characterized by meadow soils with high humus content (Kelemen et al., 2017). Three stands of mesic meadow steppes with medium intensity cattle grazing (one animal unit/ha from April to the end of July) were surveyed. Based on the dung density and on the observed forage consumption rate we can assume that the grazing intensity was similar in the three studied stands (see Oñatibia et al., 2018). We surveyed 32 plots of 0.5 m × 0.5 m size in each pasture (site); altogether there were 96 plots. We used this plot size, which was adjudged to be appropriate to accurate count of the flowering shoot numbers. Plots were designated in patches without *A. officinalis* (eight plots/pasture), and also in the vegetation with various *Althaea* cover (9–90% on site 1; 10–97% on site 2; 6–90% on site 3). The fact that the *Althaea* cover was highly variable within the sites offered an opportunity to study plots with various *Althaea* cover under the same abiotic conditions. We recorded the number of flowering shoots and cover scores of each vascular plant species in the plots in the end of July 2016.

### Data Analysis

During the statistical analyses we used linear and quadratic regression models, which included *Althaea* cover as continuous predictor. The number of flowering shoots and species number of understory species and compositional dissimilarity (measured by Bray-Curtis dissimilarity) were dependent variables. Study site was included in the models as random factor. We used two separate models for each dependent variable. Firstly, a linear mixed-effects model was fitted using a restricted maximum likelihood (REML) method (Verbeke and Molenberghs, 2009), and then we fitted another mixed-effects model with quadratic fixed-effect term (see Steiner and Leibold, 2004). We used ANOVA to compare the linear and quadratic model terms. When the ANOVA indicated non-significant difference between the two models, we kept the linear model. When ANOVA revealed significant difference between the linear and quadratic models, we kept the model characterized by the lower residual sum of squares (RSS). In case of quadratic effect terms we applied the Mitchell-Olds and Shaw test (MOS-test) to assess whether the relationship was unimodal and to obtain the location of the peak (Mitchell-Olds and Shaw, 1987).

Before model fitting we applied log-transformation on two dependent variables (number of flowering shoots and species number) using the *log(y+1)* formula (Šmilauer and Lepš, 2014). Compositional dissimilarity as a measure of small-scale heterogeneity was calculated using Bray-Curtis dissimilarity based on the species cover data. We arranged our samples within each site according to the increasing *Althaea* cover, then calculated Bray-Curtis dissimilarity for every adjacent sample pair and averaged the *Althaea* cover of these pairs. In case of samples from open pastures we calculated Bray-Curtis dissimilarity for random sample pairs. The dataset obtained from this method was appropriate for calculation of regression models. Statistical analyses were performed in R environment (R Core Team, 2017). We used the “lme” and “anova.lme” function of the “nlme” package for preparing and comparing the mixed-effects models; and “MOS-test” function for running the Mitchell-Olds and Shaw test.

## Results

**FIGURE 1:**
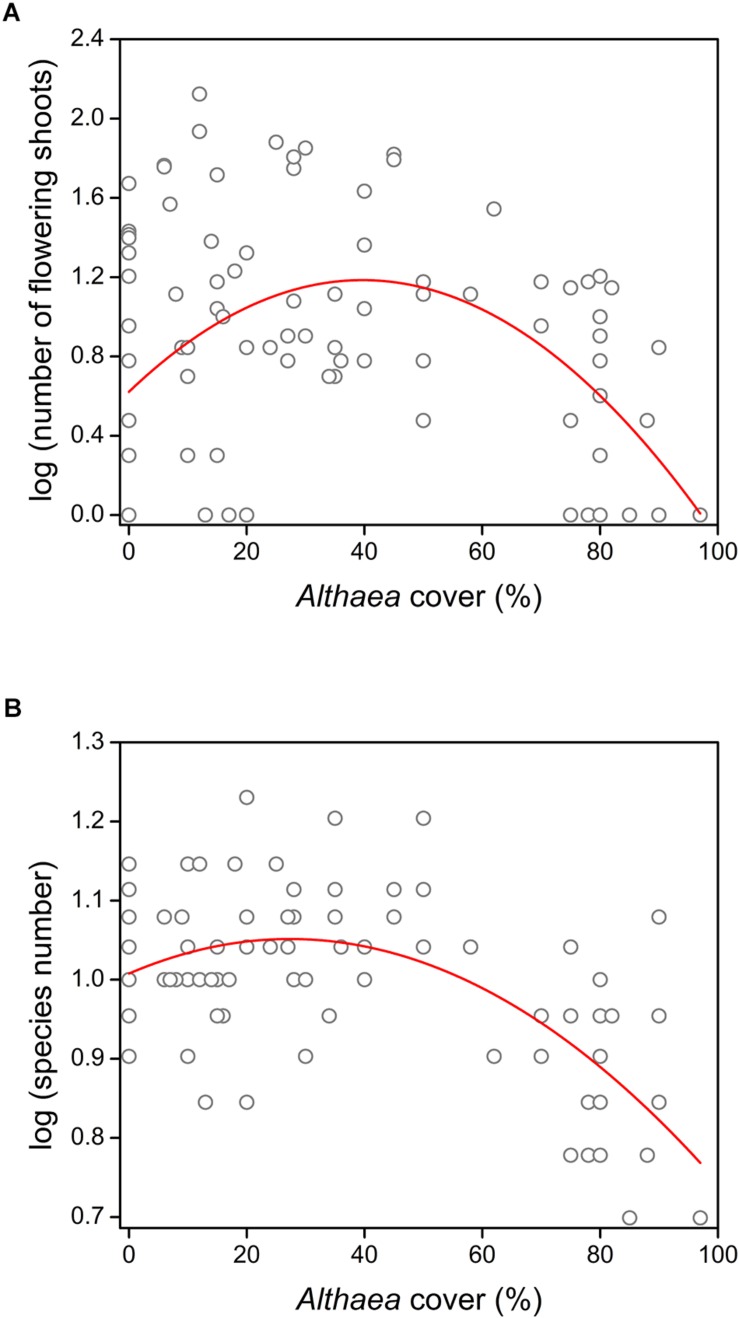
Effect of the unpalatable *Althaea officinalis* on understory species performance measures (represented in log format): **(A)** number of flowering shoots and **(B)** species number in the 0.5 m × 0.5 m plots.

**FIGURE 2:**
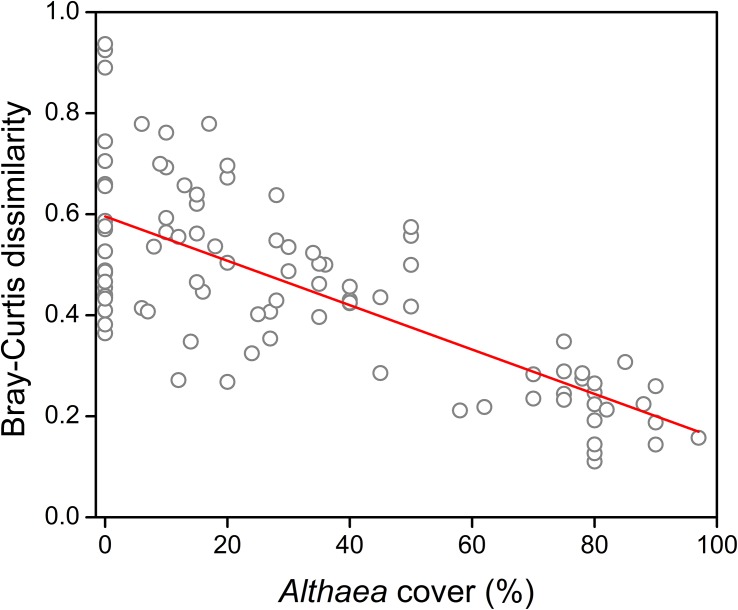
Effect of the unpalatable *Althaea officinalis* on compositional heterogeneity (Bray-Curtis dissimilarity).

We detected significant quadratic relationship between the *Althaea* cover and both the number of flowering shoots (*R*^2^ = 0.188; *F* = 10.78; *p* < 0.001) and species number (*R*^2^ = 0.378; *F* = 28.28; *p* < 0.001) (Figure 1). The results of model comparisons were the following: number of flowering shoots (RSSlinear model = 34.811; RSSquadratic model = 28.445; ANOVA: *F* = 20.82; *p* < 0.001); species number (RSSlinear model = 0.875; RSSquadratic model = 0.707; ANOVA: *F* = 22.23; *p* < 0.001). The results of MOS-tests showed that both relationships were not only curvilinear, but also unimodal (number of flowering shoots: MOS-test; *p* < 0.001; species number: MOS-test; *p* < 0.01). The peak was located at 39.7% *Althaea* cover for the number of flowering shoots and at 27.4% *Althaea* cover for the species number. Compositional dissimilarity significantly decreased with increasing *Althaea* cover and the relationship considered to be linear (*R*^2^ = 0.554; *F* = 116.9; *p* < 0.001) (Figure 2), because there was no significant difference between the linear and quadratic models (ANOVA: *F* = 1.52; *p* = 0.133). We detected non-significant site effect in all cases.

## Discussion

The relationships between the *Althaea* cover and both the number of flowering shoots and species richness were unimodal, indicating a humped-back pattern of facilitation along the density gradient of the benefactor. Most studies about the density-dependent effects of biotic refuges focused on the effects of shrub encroachment, and detected a wide variety of interactions between shrubs and herbaceous plants, including facilitation and humped-back relationship (Callaway, 2007; Kesting et al., 2015; Rolhauser and Pucheta, 2016; Saixiyala et al., 2017). Only few papers have reported facilitation between herbaceous species, because the probability of facilitation is lower among plants of similar life forms (Graff and Aguiar, 2017). Bossuyt et al. (2005) detected that the performance of palatable herbaceous species increased with increasing cover of an herbaceous benefactor in pastures. McNaughton (1978) demonstrated that the consumption of a palatable grass by bovines decreased with increasing abundance of unpalatable grasses.

In contrast with these studies, we detected humped-back pattern of understory species’ performance along the benefactor density gradient. Although grazers often avoid consuming flowering individuals (particularly graminoids), in open pastures without biotic refuges cattle repeatedly graze the palatable plants before they reach the flowering state; thus, they decrease the efficiency of generative propagation (Milchunas and Noy-Meir, 2002; Mladek et al., 2013). According to the “sequential proximity search” model, consumers try to minimize movement costs and maximize benefits of good forage quality (Acker et al., 2017; Dykes et al., 2018). Therefore, cattle generally avoid areas occupied by unpalatable species. Accordingly, the probability of palatable species being eaten decreases with the increasing density of unpalatable plants (McNaughton, 1978). In spite of this effect, the flowering success increases only in the initial part of the density gradient of benefactor, because of the increasing size-asymmetric competition by the tall, large-leaved unpalatable species at the terminal part of the gradient (Kiær et al., 2013; Kelemen et al., 2015a). The shielding decreases the available amount of light for understory species, which in turn limits plant growth and decreases photosynthetic activity (Valladares and Niinemets, 2008). Thus, flowering success declines because it highly depends on the plant size and the efficiency of photosynthesis (Bazzaz et al., 1987; Eckstein, 2005; Le Roux et al., 2013).

In perennial-dominated pastures, changes in species richness indicate the long-term effects of benefactor density and grazing. According to the “humped-back model” for the relationship between productivity and species richness, the peak of species richness is situated at intermediate productivity (Grime, 1973), and the “intermediate disturbance hypothesis” predicts the highest species richness at intermediate disturbance (Connell, 1978). *A. officinalis* is among the largest plants in the studied pastures, and grazing pressure presumably decreases with increasing benefactor density. Therefore, both the intermediate productivity and the intermediate disturbance were situated at medium benefactor density. The studied pastures are grazed by moderate intensity; therefore, the intermediate disturbance is typical not only in the patches with sparse benefactor cover but also in the open pastures. Consequently, species richness increased slightly with a gentle slope and reached its maximum at lower benefactor density (27%) than experienced in the case of flowering success (40%). The slight increment of species richness is probably also due to the long-term survival of some grazing-sensitive species sheltered by the perennial benefactor (Gibson and Brown, 1991). According to the “shifting limitations hypothesis” (see Gibson, 2009) the decline of positive interactions is expected in habitats with high density of large-sized benefactor, because of the intensifying competition (Kelemen et al., 2013; Macek et al., 2016; Goldberg et al., 2017). Intense belowground competition for nutrients and water is not expected in benign environment characterized by moist and fertile soil (Wilson and Tilman, 1991; Kiær et al., 2013); therefore, similarly to the decline of flowering success, competition for light can be responsible for the loss of species richness in the terminal part of the gradient (Lepš, 1999; Borer et al., 2014; Wang et al., 2017). Moreover, the accumulated litter and the allelopathic effect can also contribute to this phenomenon (Barkosky and Einhellig, 2003; Deák et al., 2011; Miglécz et al., 2013). The explanatory power of the models presented in this study are low, which is relatively common in researches which study animal-mediated ecological patterns, and partly due to fact that the behavior of cattle is rather unpredictable. Regardless of the low *R*^2^ values, the significant coefficients still provide reliable information about the general trends, though further studies are required to make these proposed models more precise.

The “disturbance heterogeneity model” (Kolasa and Rollo, 1991) suggests that disturbance such as moderate grazing promotes habitat heterogeneity. As an opposite effect, the encroachment of a competitive species can cause the homogenization of vegetation (Olden and Poff, 2003; Deák et al., 2015). Based on these considerations we can expect the highest small-scale heterogeneity in open pastures or at intermediate benefactor density. We detected, that compositional dissimilarity decreased with increasing benefactor density. Because of the selective grazing, in open pastures the variation in defoliation is higher than under the benefactor canopy, where the consumption reduced (see Oldén and Halme, 2016). In other words, the shelter effect smoothens or eliminates the differences between the herbivory-caused biomass loss of species in the understory vegetation. Moreover, the intensity of trampling, which plays a key role in creating spatial heterogeneity, decreases even at low unpalatable plant density (Oldén and Halme, 2016; Godó et al., 2017).

## Conclusion

In this study we detected the humped-back pattern of facilitation along the density gradient of an herbaceous unpalatable plant species in pastures. This pattern is likely based on existing conceptual models, but until now its validity has not been demonstrated for herbaceous species. It was reported only from areas affected by shrub encroachment. By the demonstration of this effect between herbaceous species we can better estimate the responses of grasslands to the encroachment of unpalatable competitors. The net effect of plant–plant interactions also depends on traits of interacting species and the studied vegetation type. Therefore, it is necessary to perform further studies in several grassland types with a wide variety of interacting species for the broad generalization of the detected patterns. Nevertheless, while planning nature conservation management it is recommended to consider retaining the sparse stands of unpalatable plants as they can positively influence the ecological functions of rangelands.

## Data Availability

All datasets for this study are included in the manuscript and the Supplementary Files.

## Author Contributions

AK, CT, OV, and BD planned the study and designed the methods. AK and CT designated the study sites. AK, CT, OV, BD, TM, and RF collected the field data. NB arranged the data. AK analyzed the data and led the writing. All authors contributed to the writing process, developed the concept of this manuscript, and reviewed the manuscript.

## Conflict of Interest Statement

The authors declare that the research was conducted in the absence of any commercial or financial relationships that could be construed as a potential conflict of interest.
